# Patient cell drug profiling identifies p53-linked vulnerabilities in refractory lymphoid malignancies

**DOI:** 10.1038/s41698-026-01630-8

**Published:** 2026-07-30

**Authors:** Arvid Cederlund, Nona Struyf, Lucia Rico Pizarro, Päivi Östling, Kristina Sonnevi, Björn Engelbrekt Wahlin, Tom Erkers

**Affiliations:** 1 Dept. of Medicine at Huddinge, Karolinska Institutet, Stockholm, Sweden; 2 Medical Unit Hematology at Solna, Cancer, Karolinska University Hospital, Stockholm, Sweden; 3 Department of Oncology-Pathology, Karolinska Institutet and Science for Life Laboratory, Stockholm, Sweden

**Keywords:** Cancer, Drug discovery, Oncology

## Abstract

Relapsed/refractory lymphoid malignancies lack effective treatment selection strategies. We evaluated high-throughput ex vivo drug profiling in 26 patients, achieving successful profiling in 22 cases. We found broad ex vivo resistance to conventional chemotherapy but sensitivity to BH3 mimetics. Notably, p53-aberrant samples showed increased sensitivity to dasatinib and PI3K inhibitors across subtypes. These findings demonstrate feasibility of functional precision medicine pipelines in lymphoid malignancies and identify actionable vulnerabilities warranting further investigation.

## Introduction

Lymphoid malignancies range from indolent lymphomas to aggressive lymphomas and acute lymphoblastic leukemia (ALL). Aggressive B-cell and T-cell lymphomas are subdivided into several entities including diffuse large B-cell lymphoma (DLBCL) and peripheral T-cell lymphoma (PTCL). Indolent disease includes mantle cell lymphoma (MCL), chronic lymphocytic leukemia and follicular lymphoma^1^.

Treatment of aggressive lymphoid malignancies is based on intensive poly(immuno-)chemotherapy protocols, although specific regimens vary by diagnosis^2^. Prognosis differs substantially across subtypes: in pediatric ALL, cure rates exceed 90%^3^, whereas real-world data indicate a cure rate of ~60% for DLBCL^4^. PTCL generally carries a poor prognosis, with only one fourth of patients achieving durable remission^5^. MCL is characterized by recurrent relapses with progressively shorter remissions and long-term remission is expected only in a minority of patients^6^. Across all aggressive lymphoid malignancies, outcomes remain poor in relapsed/refractory (R/R) disease^4–6^. New advances in treatment of R/R aggressive B-cell lymphomas and B-cell ALL (B-ALL) including chimeric antigen receptor (CAR)-T cell therapy and bispecific antibodies have led to improved outcomes, but a substantial proportion of patients relapse again^7^. Disease control prior to CAR-T cell therapy has emerged as an important determinant of outcome, a finding also observed for patients undergoing autologous hematopoietic stem cell transplantation^8,9^. These observations underscore the continued relevance of both conventional chemotherapy and novel targeted therapies, while highlighting the unmet clinical need for rational treatment selection in heavily pretreated patients.

Precision medicine aims to improve therapeutic outcomes by aligning treatment strategies with individual patient characteristics^10^. This approach is particularly appealing for heavily pretreated (R/R) patients, where prior exposure to multiple and heterogeneous regimens increases biological variability within the cohort. Early precision medicine initiatives primarily relied on genomic profiling to guide therapy selection; however, this strategy failed to identify actionable targets for a substantial proportion of patients^10^. Consequently, functional precision medicine has emerged as a complementary approach, leveraging functional assays to inform treatment decisions. Ex vivo drug sensitivity and resistance testing (DSRT) offers an opportunity to both elucidate mechanisms of drug sensitivity and resistance and may support individualized treatment selection^11,12^.

Here we present our experience and initial findings from the Drug Sensitivity Assay – Lymphoma/Leukemia (DSA-LL) observational study where a DSRT is performed on cell samples from adult patients with R/R lymphoid malignancies.

**Fig. 1:**
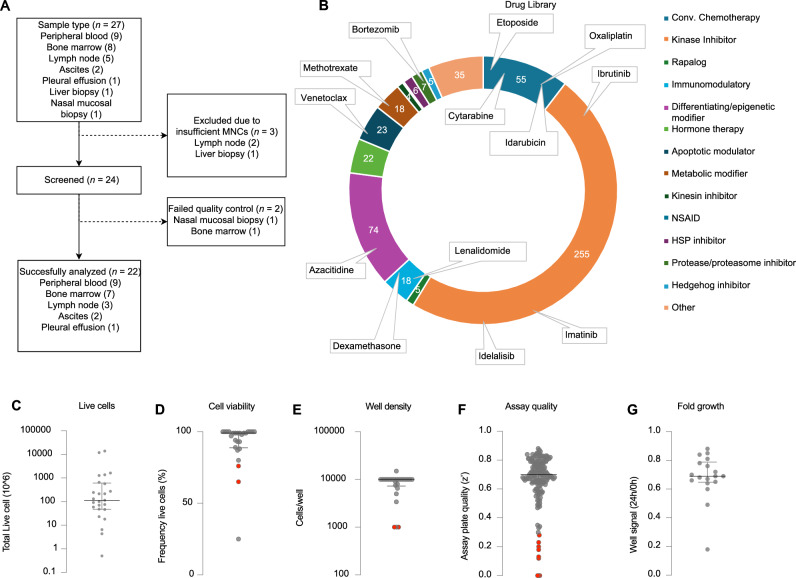
Overview of sample characteristics, drug library and assay performance. **A** Flowchart of included sample and excluded samples by tissue type. **B** Drug classes represented in the screening library; clinically relevant drugs highlighted. A full list of included drugs is presented in Supplementary Table 1. A target level breakdown of drugs in the kinase inhibitor group is presented in Supplementary Fig. 1. **C** Mononuclear cell counts per sample. **D** Pre-assay cell viability, red data points correspond to patients excluded due to failed quality control. **E** Cell density per well, red data points correspond to patients excluded due to failed quality control. **F** Assay quality assessed by Z’ factor, red data points correspond to patients excluded due to failed quality control. Values below 0 are set as 0. **G** Fold growth during 24-hour incubation, red data points correspond to patients excluded due to failed quality control. NSAID non-steroid anti-inflammatory drugs, HSP heat shock protein, MNC mononuclear cell.

To date, 26 patients have been enrolled, with one participant sampled at two different relapses. Clinical characteristics and diagnoses are summarized in Table 1. Samples were obtained from various anatomical sites (Fig. 1A). Up to 528 clinically approved and investigational drug conditions were tested in 5-point concentrations across a 10^4^-fold concentration range (Fig. 1B). A full list of included drugs is available in supplementary table 1 and a target level breakdown of included kinase inhibitors are presented in Supplementary Fig. 1. Of 27 samples, three were excluded from analysis due to insufficient mononuclear cell (MNC) yield (median cell number 9.2 × 10^6^ (range 0.01 × 10^6^–1.39 × 10^10^), Fig. 1C): all core needle biopsies. Cell viability was not a primary cause for exclusion (99% (range 25–100), Fig. 1D). Average assay well density was 10^4^ (range10^3^–10^4^) (Fig. 1E) and appears to contribute to exclusion as two samples failed assay quality control in all assay plates (median z’ 0.7 (range −33.53–0.88)) (Fig. 1F), both of which had an assay well density of 10^3^ cells/well. The metabolic activity in the samples remained relatively stable during the assay period (fold growth 0.69 (range 0.18–0.88), Fig. 1G). A majority of successfully analyzed samples, 16/22, were tested against all 528 drugs across eight plates, 5/22 samples were tested against a smaller library of 3 plates due to a lack of MNCs. One sample was tested against 7 of 8 plates due to supply issues.

**Table 1:** Overview of patient characteristics

Characteristic	Entire cohort	Successfully analyzed patients
Samples/patients (*n*)	27/26	22/21
Age (mean, range)	49.8 (18–74)	50.7 (21–74)
Male (*n*, %)	17 (63%)	14 (64%)
Female (*n*, %)	10 (37%)	8 (36%)
B-cell lymphoma (*n*, %)	13 (48%)	10 (45%)
MCL (*n*)	5	5
DLBCL (*n*)	4	3
Richter-transformation (*n*)	1	1
LBCL with 11q-aberration (*n*)	1	1
Plasmablastic lymphoma (*n*)	1	0
Hodgkin’s lymphoma	1	0
T-cell lymphoma (*n*, %)	6 (22%)	4 (18%)
PTCL-NOS (*n*)	3	2
AITL (*n*)	1	1
ALK + ALCL (*n*)	1	0
HSTCL (*n*)	1	1
Lymphoblastic disease (*n*, %)	8 (30%)	8 (36%)
Ph+ Pre-B-ALL (*n*)	2	2
Ph- Pre-B-ALL (*n*)	2	2
T-LBL or T-ALL (*n*)	4^a^	4^a^
Prior lines of therapy (median, range)	4 (1–8)	3.5 (1–8)
1 prior LoT	6	5
2 prior LoT	6	5
≥3 prior LoT	15	12
p53 aberrant (*n*, %)	6 (22%)	5 (23%)

*MCL* Mantle Cell Lymphoma, *DLBCL* Diffuse Large B-cell Lymphoma, *Ph* Philadelphia chromosome, *ALL* Acute Lymphoblastic Leukemia, *LBCL* Large B-cell Lymphoma, *PTCL-NOS* Peripheral T-cell Lymphoma No Other Specification, *AITL* Angioimmunoblastic T-cell Lymphoma, *ALCL* Anaplastic Large Cell Lymphoma, *HSTCL* Hepatosplenic T-cell Lymphoma, *LoT* Lines of Therapy.

^a^One patient included at two timepoints, at the second inclusion the disease exhibited markers similar to acute myeloid leukemia whilst maintaining the initial genetic abnormalities.

A 24-h incubation period was used for two reasons; 1) to maintain lymphoma cell viability ex vivo, as the cells exhibit a rapid decline in viability during prolonged culture^13,14^, 2) a short turnaround time is essential for future potential implementation as treatment of relapsed/refractory aggressive lymphoma and acute lymphoblastic leukemia must occur without delay.

Our findings show that ex vivo DSRT is feasible in R/R non-Hodgkin lymphoma and ALL, particularly in patients with leukemic disease or bone marrow involvement, and in those with nodal disease. Conversely, feasibility thus far has been limited in patients with exclusively solid organ involvement, although the sample size is small and further testing needs to be done.

**Fig. 2:**
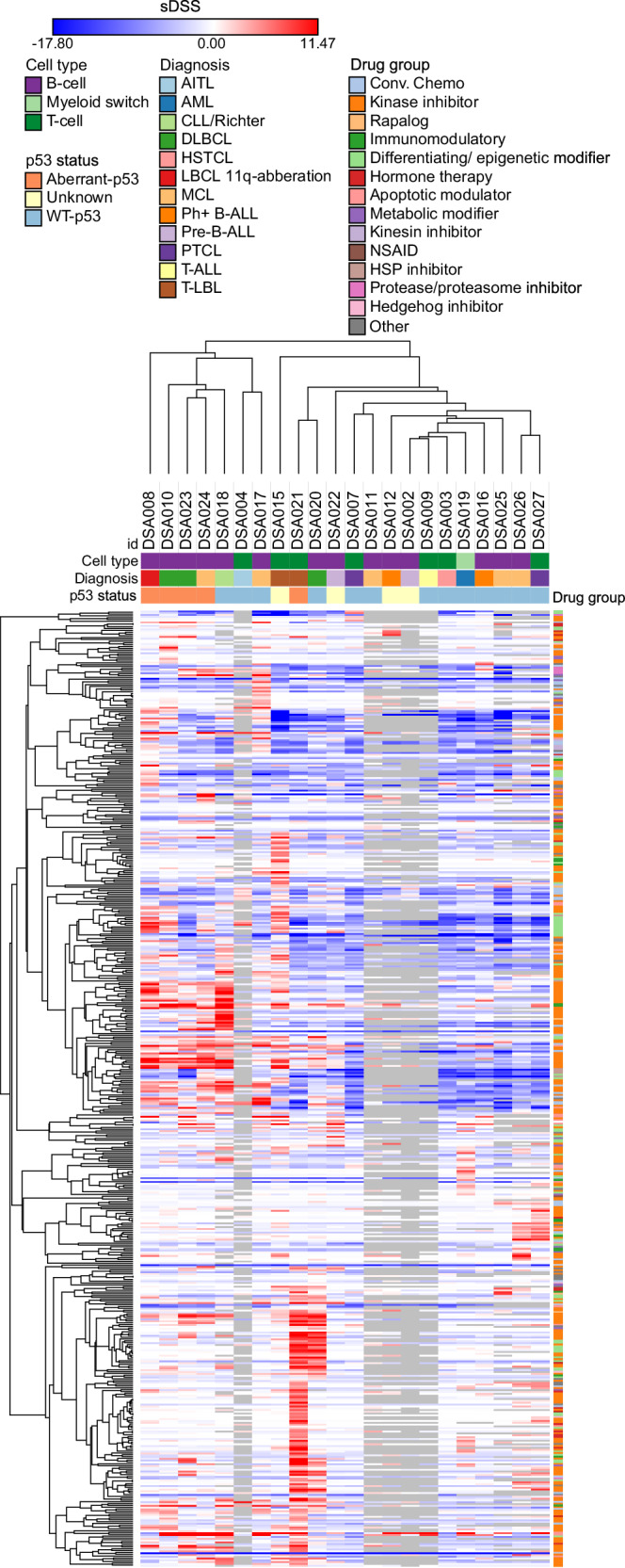
Heatmap showing the selective drug sensitivity score landscape of sampled patients. Clustering was done using 1 minus Pearson correlation. Color scale spans from the 1st percentile (dark blue) to the 99th percentile (red) with 0 as neutral (white). Gray boxes indicate drugs not tested in the specific patient sample due to 1. Insufficient cell sample for testing or 2. Unavailability of drug at the time of testing. AITL Angioimmunoblastic T-cell Lymphoma, AML Acute Myeloid Leukemia, CLL Chronic Lymphocytic Leukemia, DLBCL Diffuse Large B-cell Lymphoma, HSTCL Hepatosplenic T-cell Lymphoma, LBCL Large B-cell Lymphoma, MCL Mantle Cell Lymphoma, Ph Philadelphia chromosome, ALL Acute Lymphoblastic Leukemia, PTCL Peripheral T-cell Lymphoma, T-LBL T-Lymphoblastic Lymphoma, sDSS Selective drug sensitivity score, WT Wild type.

Ex vivo drug profiling demonstrated low selective drug sensitivity scores (sDSS^15^) for commonly used chemotherapeutic agents, including platinum compounds (oxaliplatin median sDSS −6.08, range −11.37–3.42, carboplatin median sDSS −3.77, range −3.8–7.8), nucleoside analogues (cytarabine sDSS median −6.99, range −10–6.9, fludarabine sDSS median −8.45, range −11.83–−3.7), etoposide (sDSS median −2.01, range −6.26–11.94), and anthracyclines (doxorubicin sDSS median −6, range −7.62–1, epirubicin sDSS median −6.36, range −8.85–2.22) indicating substantially lower activity compared to healthy bone marrow controls in the majority of samples. This aligns well with expectations for a cohort with extensive prior treatment^16^. Glucocorticoids such as dexamethasone and BCL-2 inhibitors such as venetoclax exhibited a range of sensitivities in our cohort (median sDSS 2.8 (range −2.5–21.6), and 2.2 (range −5.1–24.2), respectively). The BCL-2/BCL-xL inhibitor navitoclax demonstrated the most consistent sensitivity (10.0 (range −8.5–22.7)). Clinical responses to BCL-2 inhibitors have previously been reported in relapsed ALL (particularly T-ALL) and MCL^17,18^, and the success of the venetoclax-based ViPOR regimen in relapsed DLBCL^19^ supports the clinical relevance of our findings. A heatmap demonstrating sDSS values for all tested drugs is shown in Fig. 2. A heatmap displaying DSS values for all tested drugs across all included patients can be found in Supplementary fig. 2.

**Fig. 3:**
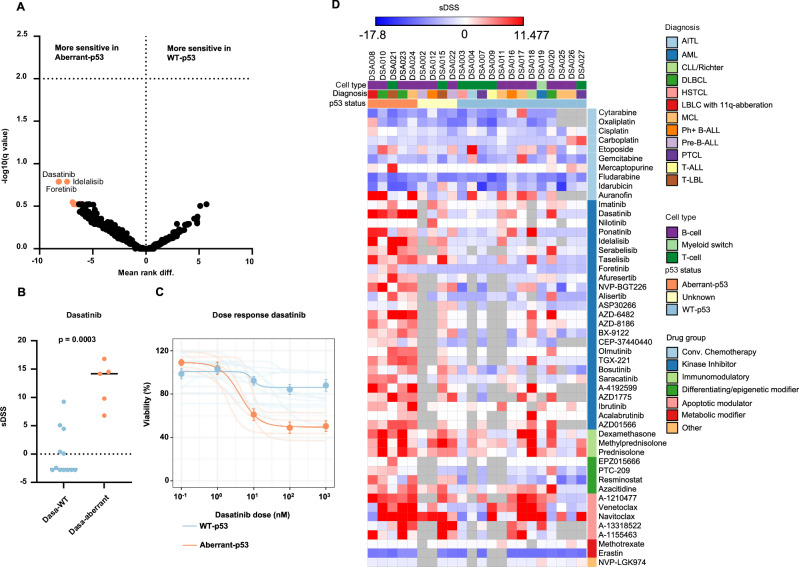
Drug sensitivity profiles and p53-associated effects. **A** Differential drug sensitivity between p53-aberrant and p53-wild-type samples using multiple Mann–Whitney U-tests (FDR = 1%); top performers based on *q*-value are highlighted as red dots and sDSS values are included figure 3D. **B** Comparison of dasatinib sDSS between p53-aberrant and p53-wild-type groups using Mann–Whitney U test. Blue dots correspond to sDSS-values from p53-wild type samples while red dots correspond to sDSS values from p53-aberrant samples. **C** Individual dose-response curves of dasatinib in representative p53-aberrant and p53-wild-type samples. Mean response curve fit across each group of patients is shown in bold. Blue curves correspond to p53-wild type samples and red curves correspond to p53-aberrant samples. **D** Heatmap showing selective drug sensitivity scores (sDSS) for a subset of 50 compounds across selected samples. Compounds were chosen based on 1. Common clinical use in relapsed/refractory disease 2. Top performance regarding sDSS (highest mean sDSS values) 3. Lowest *q*-values in multiple comparison testing or 4. BCR-ABL inhibitors and SRC-family kinase inhibitors to demonstrate differences compared to dasatinib. Color scale spans from the 1st percentile of sDSS values for all tested drugs (dark blue) to the 99th percentile of sDSS values in all samples (red) with 0 as neutral (white). Gray boxes indicate drugs not tested in the specific patient sample due to 1. Insufficient cell sample for testing or 2. Unavailability of drug at the time of testing. A table showing sDSS results in detail is available as Supplementary Data 2. AITL Angioimmunoblastic T-cell Lymphoma, AML Acute Myeloid Leukemia, CLL Chronic Lymphocytic Leukemia, DLBCL Diffuse Large B-cell Lymphoma, HSTCL Hepatosplenic T-cell Lymphoma, LBCL Large B-cell Lymphoma, MCL Mantle Cell Lymphoma, Ph Philadelphia chromosome, ALL Acute Lymphoblastic Leukemia, PTCL Peripheral T-cell Lymphoma, T-LBL T-Lymphoblastic Lymphoma, DSRT Drug screening and resistance testing, sDSS Selective drug sensitivity score, WT Wild type.

Interestingly, hierarchical clustering analysis using 1 minus Pearson correlation (Fig. 2) demonstrates a grouping of p53-aberrant, defined as TP53 mutation, del(17p), or p53 overexpression, samples. Although few risk markers overlap across these diverse diagnoses, p53 aberrations are recognized as adverse prognostic factors in most hematologic malignancies. When comparing p53-aberrant disease, to p53 wild-type (WT) disease, we observed significantly higher sDSS for dasatinib in the aberrant group compared to the wild-type group (median sDSS 14.17 vs −2.6, *P* = 0.0003, *q* = 0.162784, Supplementary Data 1). All p53-aberrant disease cases demonstrated sensitivity to dasatinib compared to one p53-WT disease case (Fig. 3A–D). Further analysis of ABL and SRC inhibitors in our dataset revealed 3 additional ABL and/or SRC inhibitors with increased activity in p53-aberrant vs p53-WT samples, saracatinib an SRC and ABL inhibitor (median sDSS 5.4 vs −1.72, *P* = 0.025372, *q* = 0.115614), bosutinib both an ABL and SRC inhibitor (median sDSS 3.75 vs −3.94, *P* = 0.028617, *q* = 0.131555) and ponatinib a ABL inhibitor (median sDSS 3.6 vs −3.68, *P* = 0.043417, *q* = 0.131555).

Sensitivity to dasatinib in p53-aberrant disease has been previously reported in CLL^20,21^. However, we observed increased dasatinib sensitivity may across multiple diagnosis in p53-aberrant R/R lymphoid malignancies. To explore this further, we leveraged publicly available data from the BEAT-AML trial^22^ to further investigate p53-aberrancy and dasatinib sensitivity in myeloid malignancies. In BEAT-AML p53-mutation was associated with decreased sensitivity to dasatinib compared to WT-p53 in AML patients (Supplementary Fig. 3A). Using DepMap^23^ to look at drug sensitivity in cancer cell lines there was no significant association between p53-mutation status and dasatinib sensitivity based on IC50 across all cancers (mutated-p53 vs unmutated-p53 median 5.535 vs 4.278, *P* = 0.3710) or in specifically lymphoid cell lines (mutated-p53 vs unmutated-p53 median 3.527 vs 2.895, *P* = 0.8274) (Supplementary Fig. 3B, C).

Furthermore, we observed a trend towards increased sensitivity to PI3K inhibitors, including Idelalisib (median sDSS 13.62 vs −2.16, *P* = 0.0009, *q* = 0.162784), Serabelisib (median sDSS 4 vs −1.54, *P* = 0.006, *q* = 0.300380), and Omipalisib (median sDSS 9.4 vs −5.75, *P* = 0.026, *q* = 0.368484). However, this was not consistent across all PI3K inhibitors.

Analysis with the present method required a large amount of MNCs limiting the applicability of our approach in Hodgkin’s lymphoma (characterized by a low proportion of Hodgkin–Reed–Sternberg cells^24^), primary CNS lymphoma and patients with limited disease burden. Reducing the number of compounds tested could improve feasibility in Hodgkin lymphoma, primary CNS lymphoma, and other cases with limited material. However, this approach restricts discovery to drugs expected to work in lymphoid malignancies, limiting repurposing opportunities. Moreover, patients with advanced disease and multiple prior therapies (median four) lack a clear standard of care, complicating design of disease-specific panels.

The limited efficacy of conventional chemotherapeutic agents in the assay requires further investigation, extensive resistance is reasonable in a heavily pre-treated cohort however other studies have shown mixed results for conventional chemotherapeutics using shorter incubations^12,25^ potentially introducing a disproportionate disadvantage for these agents.

In summary, we demonstrate that ex vivo DSRT using our approach is feasible in patients with R/R non-Hodgkin lymphoma and ALL, provided there is liquid or nodal involvement. Our findings are consistent with expectations for a heavily pretreated cohort, showing resistance to conventional chemotherapy and sensitivity to agents known to be active in R/R lymphoid malignancies, including BCL-2 inhibitors. Clinical outcome and treatment response data are being prospectively collected; future studies will evaluate their correlation with ex vivo DSRT profiles. Comprehensive mutational profiling, beyond p53 status, was not available for the current cohort. Access to broader genomic data in subsequent studies will facilitate the investigation of molecular determinants of drug resistance. The mechanistic basis for the observed dasatinib sensitivity in p53-aberrant disease remains unclear; ABL inhibition, SRC inhibition, combined target engagement, or off-target effects cannot be distinguished within this dataset, as these mechanisms are represented across multiple sensitive compounds. Larger, prospectively designed studies in heavily pretreated relapsed/refractory patients will be required to define the contributing mechanisms and establish the therapeutic relevance of this association.

## Methods

### Patient inclusion

Adult patients with R/R lymphoid malignancies were recruited from ME Hematology at Karolinska University Hospital as part of the observational study Drug Sensitivity Assay – Lymphoma/Leukemia (DSA-LL). For full inclusion and exclusion criteria see Supplementary Table 2. Cancer cell samples were taken during routine clinical sampling. All participants provided written informed consent in accordance with the Declaration of Helsinki prior to sampling and the study was approved by the Swedish Ethical Review Authority (Dnr 2020-05074 amended Dnr 2023-01527-02 and Dnr 2025-07298-02).

### Tumor cell isolation

Bone marrow and peripheral blood samples underwent MNC isolation using density gradient separation with Lymphoprep (STEMCELL Technologies, Vancouver, Canada) at 400 ×*g* for 20 min without brake. MNCs were treated with ACK lysis buffer (Thermo Fisher) for erythrocyte removal for 5 min at RT before washing the cells twice in PBS. Pleural effusion and ascites samples were centrifuged at 300 × *g* for 5 min and treated with ACK buffer if erythrocytes were present in the cell pellet. Lymph node and solid organ samples first underwent manual dissection and gentleMACS (Miltenyi Biotec, Bergish Gladbach, Germany) dissociation followed by PBS washing steps and ACK treatment in case of erythrocyte presence. Lastly, cells were sequentially strained using 70 µm and 40 µm strainers (pluriSelect, Leipzig, Germany) to remove undissociated tissue. Bone biopsy samples were first flushed with PBS using a syringe before dissociation. Finally, isolated cells were resuspended in RPMI 1640 media (Sigma-Aldrich, St. Louis, MO) containing 10% FBS (Thermo Fisher), 100 IU/mL Penicillin in combination with 0.1 mg/mL Streptomycin (Sigma-Aldrich), 2 mM L-glutamine (Sigma-Aldrich), and 12.5% HS-5 conditioned media^26^. MNCs for selected samples were then enriched using positive magnetic microbead separation (Miltenyi Biotec) based on flow cytometry results from routine clinical testing. Enrichment was performed in all cases where a distinct marker for positive selection was available.

### Drug sensitivity and resistance testing

Using a library of 528 approved and investigational oncological drugs (full list provided in supplementary table 1) on preprepared 384 multi-well plates, MNCs were added with a Multidrop reagent dispenser (Thermo Fisher) and incubated at 37 °C and 5% CO_2_ for 24 h at 5 concentration points in a 10-fold range. DMSO was used as negative control and benzethonium chloride as a positive control. After 24 h incubation viability was measured using CellTiterGlo 2.0 (Promega, Madison, WI) on an EnSight luminescence plate reader (PerkinElmer, Waltham, MA). For each sample assay quality was controlled using established metrics including Z’^27^. QC-criteria for inclusion were Z’ above 0.3 pending individual assessment of dose-response curves for drug plates with a score between 0.3 and 0.5. Drug sensitivity scores (DSS) were calculated using Breeze^28^. Before trial start, bone marrow samples from six healthy controls underwent the analysis described above. To calculate selective drug sensitivity scores (sDSS) the mean DSS values of healthy bone marrow controls were subtracted from the patient sample DSS for each patient.

### Statistics and visualizations

Initial analysis demonstrated that the data was non-normally distributed which was supported by D’Agostino–Pearson test *P*-value < 0.001. For this reason, statistical analysis was performed using multiple Mann–Whitney tests. To control for multiple comparisons, we applied a False Discovery Rate (FDR) correction using the two-stage step-up method of Benjamini, Krieger, and Yekutieli, with a desired FDR (q) set at 1%. Tests were performed using GraphPad Prism 10.6.1 (GraphPad Software, La Jolla, CA). Values of *p* < .05 were considered statistically significant. All data are displayed as median with interquartile range with all data points showed unless stated otherwise. Heatmap was generated using Morpheus (https://software.broadinstitute.org/morpheus/). Drug curves were generated with R (v.4.4.3) using RStudio (v.2024.12.1+563, Posit PBS, Boston, MA) with ggplot2 (v.4.0.0), dplyr (v.1.1.4), tidyr (1.3.1), readr (v.2.1.5), and minipack.lm (v.1.2.4).

## Supplementary information

Supplementary Information

Supplementary data 1

Supplementary data 2

## Data Availability

Data supporting the findings of this study are available from the corresponding author upon reasonable request. Patient data are subject to restricted access in accordance with the General Data Protection Regulation (GDPR), Swedish data protection laws, and the Swedish Public Access to Information and Secrecy Act. Access can be granted for projects that ensure compliance with these regulations, as outlined in a data access agreement. Once approved, data will remain accessible for the duration of the specified project.
