# Supplementary material for: Patient cell drug profiling identifies p53-linked vulnerabilities in refractory lymphoid malignancies
Source: NPJ Precis Oncol. 2026 Jul 30;10:298. doi: 10.1038/s41698-026-01630-8 (PMC13424552; doi:10.1038/s41698-026-01630-8)
Supplement: Supplementary file 1 — Supplementary Information [file 41698_2026_1630_MOESM1_ESM.pdf]

Patient Cell Drug Profiling Identifies p53-Linked Vulnerabilities in Refractory Lymphoid Malignancies - Supplementary

Supplementary table 1

ID	Drug name	Mechanism/Targets	Min conc (nM)	High.conc.(nM)
FIMM000060	Pilocarpine	Non-selective muscarinic receptor agonist	4	40000
FIMM000143	Lovastatin	HMG-CoA reductase inhibitor	1	10000
FIMM000160	Gefitinib	EGFR inhibitor	1	10000
FIMM000170	Metformin	AMPK activator	10	100000
FIMM000171	Anagrelide	PDE-3, PLA2 inhibitor	1	10000
FIMM000172	Imatinib	Abl, Kit, PDGFRB inhibitor	1	10000
FIMM000173	Idarubicin	Topoisomerase II inhibitor	0,1	1000
FIMM000176	Vatalanib	VEGFR-1 & -2 inhibitor	1	10000
FIMM000183	Erlotinib	EGFR inhibitor	1	10000
FIMM000208	Miltefosine	Antimicrobial, inhibits PI3K/AKT	10	100000
FIMM000227	Dexamethasone	Glucocorticoid, immunomodulatory agent	1	10000
FIMM000239	Auranofin	Antirheumatic agent	0,25	2500
FIMM000249	Tretinoin	Retinoic acid receptor agonist	1	10000
FIMM000255	Simvastatin	HMG CoA reductase inhibitor	1	10000
FIMM000257	Goserelin	Gonadotropin releasing hormone superagonist	1	10000
FIMM000262	Raloxifene	Selective estrogen receptor modulator	1	10000
FIMM000264	Plicamycin	RNA synthesis inhibitor	1	10000
FIMM000274	Lapatinib	HER2, EGFR inhibitor	0,1	1000
FIMM000287	Bortezomib	Proteasome inhibitor (26S subunit)	0,1	1000
FIMM000290	Letrozole	Aromatase inhibitor	1	10000
FIMM000296	Bexarotene	Antineoplastic agent; retinoid specifically selective for retinoid X receptors	1	10000
FIMM000298	Celecoxib	Selective COX-2 inhibitor	1	10000
FIMM000303	Anastrozole	Aromatase inhibitor	1	10000
FIMM000304	Bicalutamide	Nonsteriodal antiandrogen	1	10000
FIMM000321	Clofarabine	Antimetabolite; Purine analog	1	10000
FIMM000337	Vincristine	Mitotic inhibitor. Vinca alkaloid microtubule depolymerizer	0,1	1000
FIMM000344	Vinorelbine	Mitotic inhibitor. Vinca alkaloid microtubule depolymerizer	1	10000
FIMM000360	Aminoglutethimide	Anti-steroid, aromatase inhibitor	1	10000
FIMM000370	Vinblastine	Mitotic inhibitor. Vinca alkaloid microtubule depolymerizer	0,1	1000
FIMM000390	Thalidomide	Immunosuppressant	1	10000
FIMM000396	Clomifene	Selective estrogen receptor modulator	1	10000
FIMM000406	Cytarabine	Antimetabolite, interferes with DNA synthesis	1	10000,00
FIMM000406.FIMM000173	Cytarabine/Idarubicin		0,275	2750,00
FIMM000416	Disulfiram(+CuCl2)	alcohol dehydrogenase inhibitor	5	50000
FIMM000428	Finasteride	type II 5-alpha reductase inhibitor	1	10000
FIMM000439	Fluorouracil	Antimetabolite	1	10000
FIMM000446	Flutamide	Nonsteroidal antiandrogen	1	10000
FIMM000464	Imiquimod	Immunomodulatory agent, TLR7 agonist	0,25	2500

FIMM000484	Methylprednisolone	Glucocorticoid, immunomodulatory agent	1	10000
FIMM000490	Mitoxantrone	Topoisomerase II inhibitor	0,1	1000
FIMM000491	Paclitaxel	Mitotic inhibitor, taxane microtubule stabilizer	0,1	1000
FIMM000519	Prednisolone	Glucocorticoid, immunomodulatory agent	1	10000
FIMM000528	Mepacrine	Unclear. PLA2 inhibitor. NF-kB inhibitor, p53 activator	5	50000
FIMM000556	Topotecan	Topoisomerase I inhibitor. Camptothecin analog	1	10000
FIMM000560	Temozolomide	Alkylating agent	10	100000
FIMM000617	Fulvestrant	Estrogen receptor antagonist	0,1	1000
FIMM000618	Megestrol acetate	Progestogen	1	10000
FIMM000620	Tamoxifen	Estrogen receptor antagonist	1	10000
FIMM000649	Methotrexate	Antimetabolite; Anti-folate agent	0,5	5000
FIMM000773	Nilutamide	Nonsteroidal antiandrogen	1	10000
FIMM000822	Mitotane	Antineoplastic agent	1	10000
FIMM000833	Allopurinol	Xanthine oxidase inhibitor	1	10000
FIMM000914	Deferoxamine	Iron chelator	1	10000
FIMM000926	Digoxin	Cardiac glycoside	0,1	1000
FIMM000983	Hydroxyurea	Antineoplastic agent	100	1000000
FIMM001000	Mercaptopurine	Antimetabolite	1	10000
FIMM001062	Thioguanine	Antimetabolite; Purine analog	1	10000
FIMM001244	Tacrolimus	Binds FKBP12, causes inhibition of calcineurin	1	10000
FIMM001355	Epirubicin	Topoisomerase II inhibitor	0,1	1000
FIMM001376	Itraconazole	antifungal, hedgehog signaling inhibitor	0,5	5000
FIMM001823	AT 101	Bcl-2 family inhibitor	10	100000
FIMM002337	BI 2536	PLK1 inhibitor	0,1	1000
FIMM002374	SN-38	Active metabolite of irinotecan. Topoisomerase I inhibitor	1	10000
FIMM003569	Pravastatin	HMG CoA reductase inhibitor	1	10000
FIMM003593	Exemestane	Aromatase inhibitor	1	10000
FIMM003635	Raltitrexed	DHFR/GARFT/thymidylate synthase inhibitor	0,1	1000
FIMM003705	Palbociclib	CDK4/6 inhibitor	1	10000
FIMM003706	AT9283	Aurora A & B, Jak2, Flt, Abl inhibitor	0,1	1000
FIMM003707	Navitoclax	Bcl-2/Bcl-xL inhibitor	1	10000
FIMM003708	Selumetinib	MEK1/2 inhibitor	1	10000
FIMM003709	Veliparib	PARP inhibitor	1	10000
FIMM003710	Afatinib	EGFR inhibitor	0,1	1000
FIMM003711	Dovitinib	FGFR inhibitor	1	10000
FIMM003713	Crizotinib	ALK, c-Met inhibitor	0,1	1000
FIMM003714	Danusertib	Aurora, Ret, TrkA, FGFR-1 inhibitor	1	10000
FIMM003715	Foretinib	MET, VEGFR2 inhibitor	0,1	1000
FIMM003716	Abiraterone	P450 17alpha-hydroxylase-17,20-lyase inhibitor	0,5	5000
FIMM003719	SNS-032	CDK inhibitor	1	10000
FIMM003720	ABT-751	Mitotic inhibitor. Colchicine site binding microtubule depolymerizer.	1	10000
FIMM003722	BIIB021	HSP90 inhibitor	1	10000
FIMM003724	Decitabine	Nucleoside analog DNA methyl transferase inhibitor	1	10000
FIMM003725	Alvocidib	CDK inhibitor	1	10000
FIMM003728	Tipifarnib	Farnesyltransferase inhibitor	1	10000

FIMM003730	Ponatinib	Broad TK inhibitor	0,1	1000
FIMM003732	Perifosine	AKT/PI3K inhibitor	0,25	2500
FIMM003733	Motesanib	VEGFR, PDGFR, Ret, Kit inhibitor	1	10000
FIMM003734	Cediranib	KDR/Flt/VEGFR inhibitor	0,1	1000
FIMM003736	Rucaparib	PARP inhibitor	1	10000
FIMM003737	Tarenflurbil	Gamma-secretase inhibitor	1	10000
FIMM003738	Tivozanib	VEGFR1, 2, 3, c-Kit, PDGFRB inhibitor	1	10000
FIMM003739	AZD1775	Wee1 inhibitor	1	10000
FIMM003740	PF-00477736	Chk1 inhibitor	1	10000
FIMM003741	AZD7762	Chk1 inhibitor	0,1	1000
FIMM003742	AZD1152-HQPA	Aurora B inhibitor	0,1	1000
FIMM003743	AZD8055	mTOR inhibitor	1	10000
FIMM003744	Belinostat	HDAC inhibitor	1	10000
FIMM003745	Bimatoprost	Prostaglandin analog	0,55	5500
FIMM003746	Doramapimod	p38MAPK inhibitor	1	10000
FIMM003747	Bryostatin 1	PKC activator	0,01	100
FIMM003748	Tacedinaline	HDAC inhibitor	0,1	1000
FIMM003749	BMS-754807	IGF1R inhibitor	1	10000
FIMM003750	Idelalisib	PI3K inhibitor, p110?-selective	1	10000
FIMM003751	Trametinib	MEK1/2 inhibitor	0,025	250
FIMM003752	Sonidegib	Smothened (Hh) inhib	1	10000
FIMM003755	Everolimus	binds FKBP12, causes inhibition of mTORC1	0,01	100
FIMM003756	Ruxolitinib	JAK1&2 inhibitor	1	10000
FIMM003758	Indibulin	Mitoric inhibitor. Microtubule depolymerizer	1	10000
FIMM003760	MK-2206	AKT inhibitor	0,1	1000
FIMM003761	Alisertib	Aurora A inhibitor	1	10000
FIMM003762	Nelarabine	Nucleoside analog, DNA, RNA synth inhibitor	1	10000
FIMM003763	Nilotinib	Abl inhibitor	1	10000
FIMM003764	Luminespib	HSP90 inhibitor	0,1	1000
FIMM003766	Plerixafor	CXCR4 antagonist	1	10000
FIMM003767	Vemurafenib	B-Raf(V600E) inhibitor	1	10000
FIMM003769	Vandetanib	VEGFR,EGFR, RET inhibitor	0,1	1000
FIMM003771	Sepantronium bromide	Survivin inhibitor	1	10000
FIMM003772	Linsitinib	IGF1R, IR inhibitor	1	10000
FIMM003773	Tepotinib	c-Met inhibitor	0,1	1000
FIMM003775	Dasatinib	Abl, Src, Kit, EphR... Inhibitor	0,1	1000
FIMM003776	Tofacitinib	JAK3, JAK2(V617F) inhibitor	0,5	5000
FIMM003777	Fingolimod	S1PR antagonist	1	10000
FIMM003778	Axitinib	VEGFR, PDGFR, KIT inhibitor	1	10000
FIMM003779	Saracatinib	Src, Abl inhibitor	1	10000
FIMM003780	Bosutinib	Abl, Src inhibitor	1	10000
FIMM003781	Canertinib	pan-HER inhibitor	1	10000
FIMM003782	Lenalidomide	Immunomodulatory	10	100000
FIMM003783	Panobinostat	HDAC inhibitor	0,1	1000
FIMM003784	Pazopanib	VEGFR inhibitor	1	10000
FIMM003785	Tandutinib	FLT3, PDGFR, KIT inhibitor	0,1	1000

FIMM003786	Temsirolimus	binds FKBP12, causes inhibition of mTORC1	0,01	100
FIMM003787	Vorinostat	HDAC inhibitor	1	10000
FIMM003788	Entinostat	HDAC inhibitor	1	10000
FIMM003789	Enzastaurin	PKCbeta inhibitor	1	10000
FIMM003790	Olaparib	PARP inhibitor	1	10000
FIMM003791	Masitinib	KIT inhibitor	1	10000
FIMM003792	Pictilisib	PI3K inhibitor, pan-class I	1	10000
FIMM003793	Vismodegib	Smothened (Hh) inhibitor	1	10000
FIMM003794	Sorafenib	B-Raf, FGFR-1, VEGFR-2 & -3, PDGFR-beta, KIT, and FLT3 inhib	0,1	1000
FIMM003795	Seliciclib	CDK2/7/9 inhibitor	1	10000
FIMM003797	Docetaxel	Mitotic inhibitor, taxane microtubule stabilizer	0,1	1000
FIMM023794	Pentostatin	Antimetabolite; Purine analog	1	10000
FIMM023795	Sirolimus	binds FKBP12, causes inhibition of mTORC1	0,01	100
FIMM023797	Floxuridine	Antimetabolite; Analog of 5-fluorouracil	1	10000
FIMM023798	Gemcitabine	Antimetabolite; Nucleoside analog	0,1	1000
FIMM023799	Teniposide	Topoisomerase II inhibitor	1	10000
FIMM023800	Dactinomycin	RNA and DNA synthesis inhibitor	0,1	1000
FIMM023804	Cladribine	Antimetabolite; Purine analog	0,1	1000
FIMM023805	Mitomycin C	Antineoplastic anatibiotic; DNA crosslinker	1	10000
FIMM023806	Carboplatin	Platinum-based antineoplastic agent	10	100000
FIMM023807	Cisplatin	Platinum-based antineoplastic agent	10	100000
FIMM023808	Pemetrexed	Dihydrofolate reductase inhibitor	1	10000
FIMM023809	Oxaliplatin	Platinum-based antineoplastic agent	10	100000
FIMM023811	Daunorubicin	Topoisomerase II inhibitor	0,1	1000
FIMM023814	Etoposide	Topoisomerase II inhibitor	1	10000
FIMM023816	Arsenic(III) oxide	Thioredoxin reductase inhibitor; cytotoxic chemotherapeutic	0,25	2500
FIMM023818	Doxorubicin	Topoisomerase II inhibitor	0,1	1000
FIMM023819	Valrubicin	Topoisomerase II inhibitor	0,5	5000
FIMM023820	Sunitinib	Broad TK inhibitor	0,1	1000
FIMM023821	Ixabepilone	Mitotic inhibitor. Epothilone microtubule stabilizer.	0,1	1000
FIMM023825	Azacitidine	Nucleoside analog DNA methyl transferase inhibitor	1	10000
FIMM023828	Fludarabine	Antimetabolite; Purine analog	1	10000
FIMM023831	Bleomycin	Glycopeptide antibiotic; causes DNA breaks	1	10000
FIMM023832	Dactolisib	mTOR/(PI3K) inhibitor	0,1	1000
FIMM023833	Quizartinib	FLT3 inhibitor	0,1	1000
FIMM100345	APR-246	p53 activator, thioredoxin reductase 1 inhibitor	1	10000
FIMM100346	Carfilzomib	Proteasome inhibitor (20S subunit)	0,1	1000
FIMM100347	Gandotinib	JAK2 inhibitor	1	10000
FIMM100352	AZ 3146	Mps1 kinase (TTK) inhibitor	1	10000
FIMM100355	Sotrastaurin	PKC inhibitor	1	10000
FIMM100362	Midostaurin	Broad TK (FLT3, KIT, RET, JAK, EGFR...) inhibitor	1	10000
FIMM100364	Regorafenib	B-Raf, c-Kit, VEGFR2 inhibitor	1	10000
FIMM100365	XAV-939	Tankyrase-1 and -2	1	10000
FIMM100366	UCN-01	PKCbeta, PDK1, Chk, Cdk2 inhibitor	1	10000
FIMM100367	Ruboxistaurin	PKCbeta inhibitor	1	10000
FIMM100368	Capecitabine	5-FU prodrug	1	10000

FIMM100371	Chloroquine	Antimalaria agent; chemo/radio sensitizer	10	100000
FIMM100372	Valproic acid	HDAC inhibitor	100	1000000
FIMM100373	Tivantinib	MET inhibitor	0,1	1000
FIMM100374	Niraparib	PARP inhibitor	1	10000
FIMM100375	Volasertib	PLK1 inhibitor	0,1	1000
FIMM100376	Toremifene	selective estrogen receptor modulator	1	10000
FIMM100377	Lasofoxifene	Selective estrogen receptor modulator	0,1	1000
FIMM100378	Galiellalactone	STAT3-DNA interaction inhibitor	2,5	25000
FIMM100379	Omacetaxine	Protein synthesis inhib (80 S ribosome)	1	10000
FIMM100380	NVP-RAF265	"C-Raf" inhibitor, unclear MoA	0,1	1000
FIMM100382	Neratinib	HER2, EGFR inhibitor	0,1	1000
FIMM100383	Mocetinostat	HDAC inhibitor (HDAC1 & 2-selective)	1	10000
FIMM100384	Rabusertib	Chk1 inhibitor	0,1	1000
FIMM100385	Galunisertib	TGF-B/Smad inhibitor	0,1	1000
FIMM100386	Linifanib	VEGFR, PDGFR, CSF-1R, FLT3 inhibitor	0,1	1000
FIMM100387	Brivanib	VEGFR inhibitor	0,1	1000
FIMM100388	Buparlisib	PI3K inhibitor, pan-class I	1	10000
FIMM100389	Nintedanib	VEGFR, PDGFR, FGFR inhibitor	1	10000
FIMM100391	AZD4547	FGFR inhibitor	0,1	1000
FIMM100392	VER 155008	HSP70 inhibitor	1	10000
FIMM100393	Daporinad	NAMPT inhibitor	0,1	1000
FIMM100395	Cabozantinib	VEGFR2, Met, FLT3, Tie2, Kit and Ret inhibitor	0,1	1000
FIMM100396	AZD1480	JAK1/2, FGFR inhibitor	0,1	1000
FIMM100397	Ridaforolimus	binds FKBP12, causes inhibition of mTORC1	0,01	100
FIMM100398	Sonolisib	PI3K inhibitor, pan-class I. Irreversible	1	10000
FIMM100399	Quisinostat	HDAC inhibitor	0,1	1000
FIMM100402	Tosedostat	Aminopeptidase inhibitor	1	10000
FIMM100403	PF-04708671	p70S6K inhibitor	1	10000
FIMM100407	Binimetinib	MEK1/2 inhibitor	0,1	1000
FIMM100408	Tamatinib	Syk inhibitor	1	10000
FIMM100410	PF-3845	FAAH inhibitor	1	10000
FIMM100411	MK-0752	gamma-secretase/notch inhibitor	0,1	1000
FIMM100413	SB 743921	Mitotic inhibitor. Eg5/KSP inhibitor	0,01	100
FIMM100414	Omipalisib	PI3K/mTOR inhibitor	0,1	1000
FIMM100415	Ibrutinib	Btk inhibitor	0,1	1000
FIMM100416	TAK-901	Aurora, Src family, JAK3, RTK inhibitor	0,1	1000
FIMM100417	Fostamatinib	Syk inhibitor	0,25	2500
FIMM100418	JQ1	BET family inhibitor	1	10000
FIMM109440	Infigratinib	FGFR inhibitor	0,1	1000
FIMM109441	Momelotinib	JAK1 & 2 inhibitor	1	10000
FIMM109442	Sapanisertib	mTOR inhibitor	0,1	1000
FIMM109444	TGX-221	PI3K beta selective inhibitor	1	10000
FIMM109445	Tubacin	HDAC6 inhibitor	1	10000
FIMM109446	Tubastatin A	HDAC6 inhibitor	1	10000
FIMM109447	Atorvastatin	HMG CoA reductase inhibitor	1	10000
FIMM109448	Varespladib	Secretory phospholipase A2 inhibitor	1	10000

FIMM109449	StemRegenin 1	AHR antagonist, stem cell regenerating	1	10000
FIMM109451	PFI-1	BET family inhibitor	3	30000
FIMM109453	4-hydroxytamoxifen	Selective estrogen receptor modulator	1	10000
FIMM109456	Enzalutamide	AR antagonist	1	10000
FIMM109458	1-methyl-D-tryptophan	Indolamine 2,3-dioxygenase 1 and 2 inhibitor	0,5	5000
FIMM109459	Dacomitinib	pan-HER inhibitor	0,1	1000
FIMM109460	Dinaciclib	CDK inhibitor	0,1	1000
FIMM109461	Dabrafenib	B-Raf(V600E) inhibitor	0,25	2500
FIMM109462	I-BET151	BET family inhibitor	1	10000
FIMM109463	Ralimetinib	p38MAPK inhibitor	1	10000
FIMM109464	Crenolanib	PDGFRA and PDGFRB inhibitor	1	10000
FIMM109465	PF-4800567	CK1epsilon inhibitor	1	10000
FIMM109467	PF-670462	CK1epsilon and CK1delta inhibitor	1	10000
FIMM109468	ZSTK474	PI3K gamma selective inhibitor	1	10000
FIMM115468	PAC-1	procaspase-3 activator	1	10000
FIMM115469	Vistusertib	mTOR inhibitor, ATP-competitive	1	10000
FIMM115470	CPI-613	pyruvate dehydrogenase, alpha-ketoglutarate dehydrogenase inhibitor	1	10000
FIMM115471	Roxadustat	HIF prolyl hydroxylase inhibitor	1	10000
FIMM115472	Pevonedistat	NAE inhibitor	1	10000
FIMM115473	GSK2636771	PI3K beta selective inhibitor	1	10000
FIMM115474	AT-406	XIAP, cIAP1, cIAP2 inhibitor	1	10000
FIMM115478	Lomeguatrib	O6-methylguanine-DNA methyltransferase inhibitor	1	10000
FIMM115479	GSK269962	ROCK1 and ROCK2 inhibitor	1	10000
FIMM115482	GSK650394	SGK1 & 2 inhibitor	1	10000
FIMM115483	AVN944	IMPDH inhibitor	1	10000
FIMM115484	Venetoclax	Bcl-2-selective inhibitor	0,1	1000
FIMM115485	BMS-911543	JAK2 inhibitor	1	10000
FIMM115486	Ipatasertib	AKT inhibitor	1	10000
FIMM115606	IOX-2	PHD2 inhibitor	5	50000
FIMM115607	GSK-J4	JMJD3 (histone demethylase) inhibitor	10	100000
FIMM115608	UNC1215	L3MBTL3 inhibitor	1	10000
FIMM115609	SGC0946	DOT1L inhibitor	1	10000
FIMM115610	UNC0642	G9a/GLP inhibitor	1	10000
FIMM115611	GSK343	EZH2 inhibitor	0,1	1000
FIMM115612	UNC0638	G9a/GLP inhibitor	1	10000
FIMM115613	C646	p300/CREB-binding protein (CBP) inhibitor	2,5	25000
FIMM115614	8-chloro-adenosine	Nucleoside analog, RNA synthesis inhibitor	5	50000
FIMM115615	8-amino-adenosine	Nucleoside analog, RNA synthesis inhibitor	5	50000
FIMM133776	Rocilinostat	HDAC-6 selective inhibitor	1	10000
FIMM133777	ASP3026	ALK inhibitor	1	10000
FIMM133778	Alectinib	ALK (incl gatekeeper mut) inhib	0,1	1000
FIMM133782	Ganetespib	HSP90 inhibitor	0,1	1000
FIMM133783	TRAM-34	intermediate-conductance Ca2+-activated K+ channel inh.	0,1	1000
FIMM133784	Pracinostat	HDAC inhibitor	1	10000

FIMM133785	AR-42	HDAC inhibitor	1	10000
FIMM133786	Lonafarnib	Farnesyl transferase inhibitor	10	100000
FIMM133787	Alpelisib	PI3Kalpha selective inhibitor	0,25	2500
FIMM133788	Baricitinib	JAK inhibitor	0,25	2500
FIMM133789	BX-912	PDK1 inhib	1	10000
FIMM133791	AZD-5363	AKT inhibitor	1	10000
FIMM133792	CUDC-305	HSP90 inhibitor	1	10000
FIMM133793	Oprozomib	proteasome (20 S) inhibitor	0,25	2500
FIMM133794	Fedratinib	JAK2-selective inhibitor	1	10000
FIMM133795	PF-03758309	PAK inhibitor	1	10000
FIMM133796	GSK-2334470	PDK1 inhibitor	1	10000
FIMM133797	Pomalidomide	Immunomodulatory agent, anti-angiogenic	1	10000
FIMM133798	Bafetinib	Abl, Lyn inhibitor	0,1	1000
FIMM133799	Orteronel	CYP17A1, androgen synth inhib.	1	10000
FIMM133802	EPZ-5687	EZH2 inhibitor	1	10000
FIMM133803	LY-2874455	FGFR inhibitor	0,1	1000
FIMM133804	Apalutamide	AR antagonist	1	10000
FIMM133805	Tideglusib	GSK3 inhibitor	0,3	3000
FIMM133806	SGI-1776	PIM kinase inhibitor	1	10000
FIMM133807	Rigosertib	Ras-Raf interaction inhibitor, contaminated by microtubile depolymerizer...	1	10000
FIMM133808	Milciclib	CDK2 inhibitor	1	10000
FIMM133809	Lenvatinib	VEGFR inhibitor	0,25	2500
FIMM133810	Duvelisib	PI3K inhibitor	0,05	500
FIMM133811	Icotinib	EGFR inhibitor	1	10000
FIMM133812	Cilengitide	alphaVbeta3 integrin inhibitor	1	10000
FIMM133813	Apatinib	VEGFR inhibitor	1	10000
FIMM133815	Amuvatinib	Broad spectrum TK inhib	1	10000
FIMM133816	SCH772984	ERK1 & 2 inhibitor	1	10000
FIMM133819	Telatinib	VEGFR, KIT, PDGFR inhibitor	1	10000
FIMM133820	Triciribine	AKT inhibitor	10	100000
FIMM133821	Tozasertib	pan-Aurora inhibitor	1	10000
FIMM133822	Varlitinib	EGFR HER2 inhibitor	1	10000
FIMM133823	Golvatinib	MET, VEGFR2 inhibitor	0,25	2500
FIMM133824	Copanlisib	PI3K alpha, delta selective inhibitor	0,1	1000
FIMM133825	Sapitinib	Pan-HER inhibitor	0,1	1000
FIMM133827	AT7519	CDK1, 2, 4, 6 and 9 inhibitor	1	10000
FIMM133828	NVP-BGT226	PI3K/mTOR inhibitor	0,1	1000
FIMM133829	BMS-777607	Met, Axl, Ron and Tyro3 inhibitor	0,25	2500
FIMM133830	Abemaciclib	CDK4/6 inhibitor	0,25	2500
FIMM133831	Ceritinib	ALK inhibitor	0,25	2500
FIMM133834	Neflamapimod	p38MAPK inhibitor	1	10000
FIMM133835	Tesevatinib	EGFR, ERBB2, VEGFR, EPHB4	0,1	1000
FIMM133837	Hydroxyfasudil	ROCK, PKA, PKG, PRK inhibitor	1,9	19000
FIMM133838	Lucitanib	FGFR1, VEGFR inhibitor	1	10000
FIMM133841	ENMD-2076	pan-Aurora, VEGFR inhibitor	1	10000
FIMM133843	PD0325901	MEK1/2 inhibitor	0,1	1000

FIMM133844	PH-797804	p38MAPK inhibitor	0,1	1000
FIMM133846	TG100-115	PI3K gamma/delta inhibitor	1	10000
FIMM133849	Tucatinib	HER2 inhibitor	0,25	2500
FIMM133850	GSK-1070916	AURb, AURc inhibitor	0,1	1000
FIMM133851	GSK-461364	PLK1 inhibitor	1	10000
FIMM133852	Capmatinib	MET inhibitor	0,1	1000
FIMM133854	Palomid-529	AKT, MTOR, PI3K inhibitor	1	10000
FIMM133855	PF-00562271	FAK inhibitor	1	10000
FIMM133857	Gedatolisib	PI3K/mTOR inhibitor	0,1	1000
FIMM133859	TAK-285	HER2 inhibitor	0,25	2500
FIMM133860	CEP-32496	BRAF inhibitor	1	10000
FIMM133862	IOX-1	2-Oxoglutarate Oxygenase Inhibitor	10	100000
FIMM133870	AZD-6482	PI3Kbeta-selective inhibitor	0,25	2500
FIMM133876	GSK-690693	AKT, PKA, PKC inhibitor	1	10000
FIMM133877	OSU-03012	PDPK1 inhibitor	2,5	25000
FIMM133878	NVP-AEW541	IGF1R inhibitor	1	10000
FIMM133880	AZD-1080	GSK3 inhibitor	1	10000
FIMM133882	MK-8776	CHEK1 inhibitor	0,25	2500
FIMM133885	AZD-5438	CDK1,2,9 inhibitor	1	10000
FIMM133886	Silmitasertib	CSNK2A1 inhibitor	1	10000
FIMM133887	Mubritinib	HER2 inhibitor	0,1	1000
FIMM133888	AZD-8186	PI3Kbeta inhibitor	0,1	1000
FIMM133889	GDC-0623	MEK1/2 inhibitor	0,25	2500
FIMM133890	Talmapimod	p38MAPK alpha selective inhibitor	1	10000
FIMM133891	Birinapant	IAPs, SMAC mimetic	0,1	1000
FIMM133892	NVP-LCL161	IAPs, SMAC mimetic	2,5	25000
FIMM133893	Tazemetostat	EZH2 inhibitor	1	10000
FIMM133896	Pinometostat	DOT1L inhibitor	0,1	1000
FIMM133897	PHA 408	IKK-2 inhibitor	1	10000
FIMM133898	PS-1145	IKK-2 inhibitor	2,5	25000
FIMM133899	KU-60019	ATM inhibitor	2,5	25000
FIMM133902	Selinexor	XPO1/CRM1 inhibitor	1	10000
FIMM133904	GSK2801	BAZ2B/A bromodomain inhibitor	1	10000
FIMM133905	SGC-CBP30	CREBBP/EP300 bromodomain inhibitor	2,5	25000
FIMM136357	TH588	MTH1 inhibitor	2,5	25000
FIMM136365	VGX-1027	Nitric oxide-donating immunomodulator	1	10000
FIMM136366	Birabresib	BET family inhibitor	1	10000
FIMM136367	Encorafenib	B-Raf(V600E) inhibitor	0,1	1000
FIMM136368	URB597	FAAH inhibitor	0,1	1000
FIMM136369	CUDC-907	HDAC1/2/3/10, PI3Kalpha inhibitor	1	10000
FIMM136370	MK-8745	Aurora A inhibitor	1	10000
FIMM136371	GDC-0919	IDO inhibitor	1	10000
FIMM136372	NVP-LGK974	PORCN inhibitor	1	10000
FIMM136373	ONX-0914	LMP7 (immunoproteasome)	1	10000
FIMM136374	RGFP966	HDAC3 inhibitor	1	10000
FIMM136375	NMS-873	p97/VCP inhibitor	1	10000

FIMM136376	AZ191	DYRK1A inhibitor	1	10000
FIMM136377	Ribociclib	CDK4/6 inhibitor	1	10000
FIMM136378	Triapine	ribonucleotide reductase inhibitor	1	10000
FIMM136379	VE-821	ATR inihibitor	1	10000
FIMM136380	Pacritinib	FLT3/JAK2	1	10000
FIMM136381	Tanzisertib	JNK inhibitor	1	10000
FIMM136382	GSK923295	CENP-E inhibitor	1	10000
FIMM136383	Bentamapimod	JNK inhibitor	1	10000
FIMM136384	Idasanutlin	p53-MDM2 inhibitor	1	10000
FIMM136385	Aldoxorubicin	Topoisomerase II inhibitor, Albumin binding	0,1	1000
FIMM136386	Filanesib	KSP/Eg5 inhibitor	0,1	1000
FIMM136388	Talazoparib	PARP1/2 inhibitor	0,1	1000
FIMM136389	Tasquinimod	S100A9 inhibitor, immunomodulatory, anti-angiogenic	1	10000
FIMM136390	Resminostat	HDAC1, 3, 6 inhibitor	1	10000
FIMM136391	WEHI-539	Bcl-XL inhibitor	0,25	2500
FIMM136392	Cobimetinib	MEK1/2 inhibitor	0,1	1000
FIMM136393	AZD1208	PIM1, 2, 3 kinase inhibitor	1	10000
FIMM136394	BGB324	Axl inhibitor	1	10000
FIMM136397	AZD7545	PDHK inhibitor	1	10000
FIMM136398	Osimertinib	EGFR(L858R/T790M) inhibitor	0,25	2500
FIMM136399	BMS863233	Cdc7 inhibitor	1	10000
FIMM136400	Darapladib	lipoprotein-associated phospholipase A2 inhibitor	0,1	1000
FIMM136401	GNE-0877	LRRK2 inhibitor	0,1	1000
FIMM136402	Entospletinib	SYK inhibitor	0,5	5000
FIMM136403	Ixazomib	20S proteasome inhibitor	0,1	1000
FIMM136404	Losmapimod	p38MAPK inhibitor	1	10000
FIMM136405	ML323	USP1-UAF1 inhibitor	1	10000
FIMM136406	OTS167	MELK inhibitor	0,1	1000
FIMM136407	PTC-209	BMI-1 inhibitor	1	10000
FIMM136409	UNC2881	MER inhibitor	0,25	2500
FIMM136412	AMG-232	MDM2 inhibitor	1	10000
FIMM136413	AMG-925	FLT-3, CDK4 inhibitor	0,1	1000
FIMM136414	CEP-37440	ALK inhibitor	0,5	5000
FIMM136415	Rociletinib	EGFR(L858R/T790M) inhibitor	1	10000
FIMM136416	FRAX486	PAK1, 2, 3 inhibitor	0,5	5000
FIMM136417	GNE-7915	LRRK2 inhibitor	0,1	1000
FIMM136418	GSK2830371	Wip1 inhibitor	0,5	5000
FIMM136419	Molibresib	BET family inhibitor	1	10000
FIMM136426	BCI	Dusp6 inhibitor	5	50000
FIMM136427	MST-312	Telomerase inhibitor	1	10000
FIMM136428	Taselisib	PI3K alpha, delta, (gamma) selective inhibitor	0,1	1000
FIMM136429	GSK2656157	PERK inhibitor	0,25	2500
FIMM136430	TAK-530	pan-RAF inhibitor	1	10000
FIMM136431	Glasdegib	Smo inhibitor	0,1	1000
FIMM136434	Onalespib	HSP90 inhibitor	0,25	2500
FIMM136436	Uprosertib	AKT inhibitor	1	10000

FIMM136437	AT13148	p70S6K, PKA, ROCK (AKT) inhibitor	1	10000
FIMM136438	Cerdulatinib	JAK, SYK inhibitor	1	10000
FIMM136440	Filgotinib	JAK1-selective inhibitor	1	10000
FIMM136448	Afuresertib	AKT1-selective inhibitor	0,1	1000
FIMM136449	UM729	Enhancer of aryl hydrocarbon receptor (AhR) antagonists	1	10000
FIMM136450	PF-06463922	ALK, ROS1 inhibitor	0,1	1000
FIMM136452	TEW-7197	TGF-? receptor ALK4/ALK5 inhibitor	0,25	2500
FIMM136453	Sabutoclax	pan-Bcl-2 family inhibitor	2,5	25000
FIMM136456	SH-4-54	STAT3 inhibitor	2,5	25000
FIMM136458	BAY 87-2243	HIF1alpha inhibitor	0,1	1000
FIMM136465	Givinostat	HDAC inhibitor	0,1	1000
FIMM136466	Amsacrine	DNA intercalation, Topo II inhibitor	1	10000
FIMM136467	PCI-34051	HDAC8 inhibitor	1	10000
FIMM136469	Cabazitaxel	Taxane microtubule stabilizer, antimitotic	0,1	1000
FIMM136470	Ravoxertinib	ERK inhibitor	1	10000
FIMM136472	KD025	ROCK2 inhibitor	0,5	5000
FIMM136473	Merestinib	Met inhibitor	0,1	1000
FIMM136474	Pixantrone	topoisomerase II inhibitor	1	10000
FIMM136475	Poziotinib	pan-HER inhibitor	0,1	1000
FIMM136477	Spebrutinib	BTK inhibitor	0,1	1000
FIMM136478	Verdinexor	XPO1/CRM1 inhibitor	0,1	1000
FIMM136480	Romidepsin	HDAC inhibitor	0,1	1000
FIMM136481	Abexinostat	HDAC1-selective inhibitor	1	10000
FIMM136488	A-1210477	MCL-1 inhibitor	5	50000
FIMM136490	VS-4718	FAK inhibitor	1	10000
FIMM136491	GSK2879552	LSD1 inhibitor	10	100000
FIMM136493	Ulixertinib	ERK inhibitor	1	10000
FIMM136494	LY3009120	pan-RAF inhibitor	1	10000
FIMM136495	Pexidartinib	KIT, CSF1R, FLT3 inhibitor	1	10000
FIMM136496	Marimastat	MMP-9, MMP-1, MMP-2, MMP-14, MMP-7 inhibitor	1	10000
FIMM136497	EPZ015666	PRMT5 inhibitor	1	10000
FIMM136498	SAR405838	MDM2 inhibitor	1	10000
FIMM136500	Prexasertib	Chk1 inhibitor	1	10000
FIMM136501	Epacadostat	IDO inhibitor	1	10000
FIMM136502	AZD3965	MCT1 inhibitor	0,1	1000
FIMM136503	Decernotinib	JAK3 inhibitor	1	10000
FIMM136504	Omaveloxolone	Nrf2 activator	1	10000
FIMM136505	BRD7116	Leukemic stem cell inhibitor	1	10000
FIMM136506	A-419259	HCK and other SRC family kinase inhibitor	1	10000
FIMM136507	Napabucasin	CSC inhibitor, STAT3 mediated	2	20000
FIMM136508	Enasidenib	IDH2-R140Q inhibitor	1	10000
FIMM136509	CCT196969	pan-RAF/Src inhibitor	2,5	25000
FIMM136510	Gilteritinib	FLT3/AXL inhibitor	0,1	1000
FIMM136511	ARV-825	BET-targeting PROTAC	0,03	300
FIMM136512	dBET1	BET-targeting PROTAC	1	10000
FIMM136513	A-1331852	Bcl-XL inhibitor	0,1	1000

FIMM136514	A-1155463	BCL-XL inhibitor	1	10000
FIMM136515	Ivosidenib	IDH1 R132H/R132C inhibitor	1	10000
FIMM136516	Peficitinb	JAK3-selective inhibitor	0,25	2500
FIMM136517	DEL-22379	ERK dimerization inhibitor	5	50000
FIMM136518	AMG319	PI3Kdelta inhibitor	0,1	1000
FIMM136519	TIC10	ERK & AKT inhibitor, TRAIL inducer	2,5	25000
FIMM136520	ABC294640	Sphingosine kinase 2 inhibitor	5	50000
FIMM136521	Radotinib	ABL, PDGFR inhibitor	1	10000
FIMM136522	Ripasudil	ROCK inhibitor	1	10000
FIMM136523	CC-223	mTOR inhibitor	1	10000
FIMM136524	CC-115	mTOR/DNA-PK inhibitor	1	10000
FIMM136525	OTS-964	TOPK inhibitor	0,25	2500
FIMM136526	RO5126766	dual RAF/MEK inhibitor	0,1	1000
FIMM136527	TGR-1202	PI3Kdelta inhibitor	0,25	2500
FIMM136528	Litronesib	Eg5 inhibitor	0,1	1000
FIMM136529	AZD6738	ATR inihibitor	2,5	25000
FIMM136530	ODM-201	AR antagonist	0,25	2500
FIMM136531	NVP-CGM097	p53-MDM2 inhibitor	2,5	25000
FIMM136532	Entrectinib	TRK, ROS1, ALK inhibitor	0,1	1000
FIMM136533	LY3023414	PI3K/mTOR/DNA-PK inhibitor	0,25	2500
FIMM136534	Tirabrutinib	BTK inhibitor	0,1	1000
FIMM136535	Resatorvid	TLR4 inhibitor	1	10000
FIMM136536	Resiquimod	TLR7/TLR8 agonist	1	10000
FIMM136537	Motolimod	TLR8 agonist	1	10000
FIMM136538	THZ2	CDK7 inhibitor	1	10000
FIMM136539	Glesatinib	MET, AXL, TIE, VEGFR, RON inhibitor	0,25	2500
FIMM136540	Sitravatinib	RET, TRK, PDGFR, VEGFR, KIT, DDR... inhibitor	0,25	2500
FIMM136547	BGB-283	Raf inhibitor	1	10000
FIMM136548	Asciminib	Allosteric ABL inhibitor, blocks myristoyl binding	0,1	1000
FIMM136549	Eribulin	Mitotic inhibitor, microtubule depolymerizer.	0,1	1000
FIMM136551	Brigatinib	ALK inhibitor, including gatekeeper mutant ALK	0,1	1000
FIMM136552	Selonsertib	ASK1 inhibitor	0,1	1000
FIMM136553	Pirfenidone	Antifibrotic and anti-inflammatory	1	10000
FIMM136554	CPI-0610	BET family inhibitor	1	10000
FIMM136555	CPI-360	EZH2 inhibitor	1	10000
FIMM136556	Serabelisib	PI3Kalpha selective inhibitor	1	10000
FIMM136557	NVP-SHP099	SHP2 inhibitor	1	10000
FIMM136559	Olmutinib	EGFR(L858R/T790M) inhibitor	0,1	1000
FIMM136561	Altiratinib	MET/Tie-2 inhibitor	1	10000
FIMM136562	AZD0156	ATM inihibitor	0,1	1000
FIMM136563	Acalabrutinib	BTK inhibitor	0,1	1000
FIMM136564	Trifluridine	Antimetabolite; Nucleoside analog	1	10000
FIMM136565	AZD3759	EGFR inhibitor, BBB penetrable	0,1	1000
FIMM136566	Salinomycin	Ionophore	5	50000
FIMM136567	Mivebresib	BET family inhibitor	1	10000
FIMM136568	VLX1570	proteasome deubiquitinase inhibitor	1	10000

FIMM136569	A-366	G9a/GLP inhibitor	2,5	25000
FIMM136571	LY-2584702	p70S6K inhibitor	1	10000
FIMM136572	Taladegib	Smothened (Hh) inhib	1	10000
FIMM136573	Acitretin	Retinoid receptor agonist	1	10000
FIMM136574	NVP-BHG712	EphB4 inhibitor	1	10000
FIMM136575	Necrostatin 2	Necroptosis inhibitor	1	10000
FIMM136576	Tucidinostat	HDAC1/2/3/10 inhibitor	1	10000
FIMM136577	Vesatolimod	TLR7 agonist	1	10000
FIMM136578	AMG-337	Met inhibitor	1	10000
FIMM136579	Vidofludimus	DHODH inhibitor	1	10000
FIMM136580	ML390	DHODH inhibitor	5	50000
FIMM136581	E7820	Integrin alpha2 expression inhibitor	5	50000
FIMM136582	JPH203	LAT1 inhibitor	0,5	5000
FIMM136583	GDC-0084	PI3K/mTOR inhibitor	1	10000
FIMM136584	PF06650833	IRAK4 inhibitor	1	10000
FIMM136585	CC122	IMiD immunomodulator	1	10000
FIMM136586	Amcasertib	Cancer stem cell kinase inhibitor	1	10000
FIMM136587	Eltanexor	XPO1/CRM1 inhibitor	1	10000
FIMM136588	PIM-447	PIM1, 2, 3 kinase inhibitor	1	10000
FIMM136589	EPZ031686	SMYD3 inhibitor	1	10000
FIMM136590	Vinflunine	Mitotic inhibitor. Vinca alkaloid microtubule depolymerizer	0,1	1000
FIMM136591	Ensartinib	ALK inhibitor	0,1	1000
FIMM136592	GSK2256098	FAK inhibitor	0,1	1000
FIMM136593	GDC-0853	BTK inhibitor	0,1	1000
FIMM136594	Erdafitinib	FGFR inhibitor	0,1	1000
FIMM136595	Larotrectinib	TRK inhibitor	0,1	1000
FIMM136596	Senexin B	CDK8/19 inhibitor	0,1	1000
FIMM136597	BAY-1436032	IDH1 R132H/R132C inhibitor	1	10000
FIMM136598	Saridegib	Smothened (Hh) inhib	1	10000
FIMM136599	Upadacitinib	JAK1-selective inhibitor	1	10000
FIMM136600	S-63845	MCL-1 inhibitor	0,1	1000
FIMM136615	Erastin	VDAC inhibitor, induces ferroptosis	1	10000
FIMM136616	RSL3	GPX4 inhibitor, induces ferroptosis	1	10000

Full drug library used for drug sensitivity and resistance testing including doses. Min and max concentrations are shown in columns 4 and 5 respectively. All drugs were tested on a 10000-fold concentration range at 5 evenly distributed points (i.e. 1, 10, 100, 1000 and 10000) between the minimum and maximum concentration.

Supplementary table 2

Inclusion	Exclusion
Age ≥ 18 years	Psychiatric disorder or dementia which make the patient unable to give informed consent
Relapsed or refractory lymphoma or leukemia	
Measurable disease defined as:<br>- At least one two-dimensionally measurable lesion with a longest diameter > 15 mm<br>OR<br>- Leukemic cells detectable by flow cytometry or standard cell counts in bone marrow or peripheral blood	
WHO performance status 0-4	

Full inclusion and exclusion criteria for drug sensitivity assay lymphoma/leukemia (DSA-LL).

Supplementary figure 1

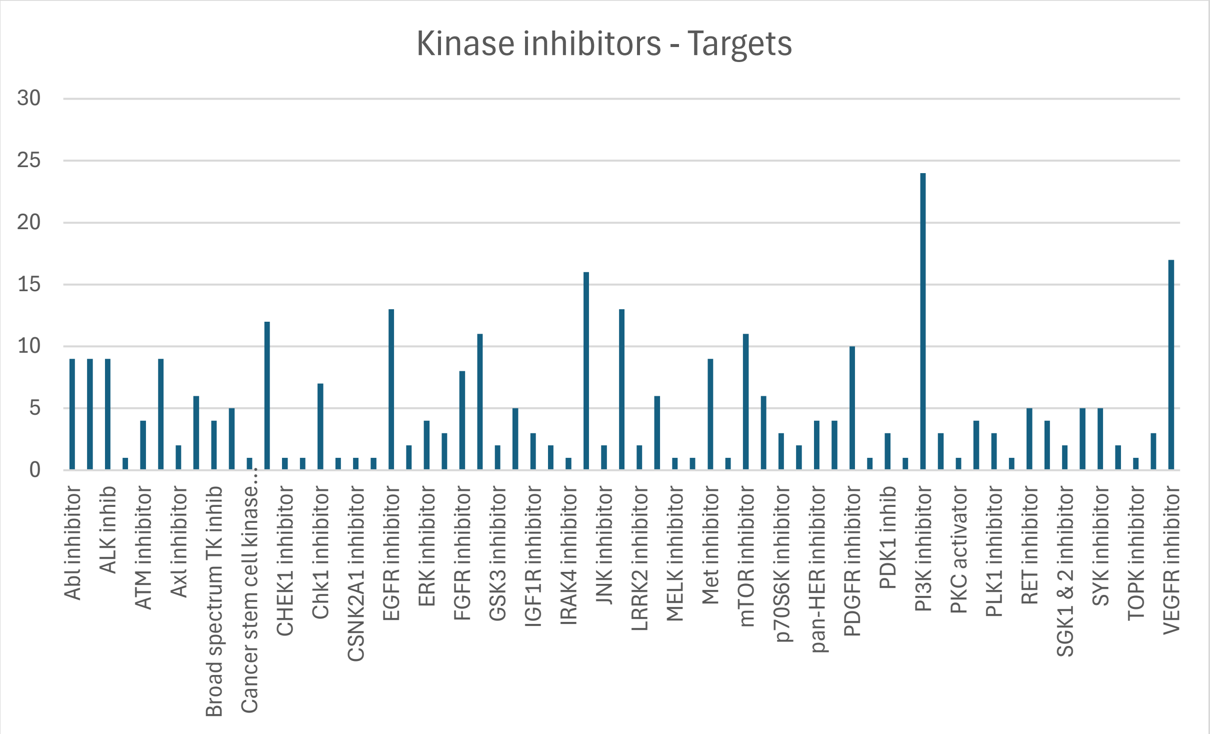

Bar graph displaying the targets of drugs in group B – kinase inhibitors in our drug library. Note that the sum is greater than the number of drugs in group B as some inhibitors cover multiple targets.

Supplementary figure 2

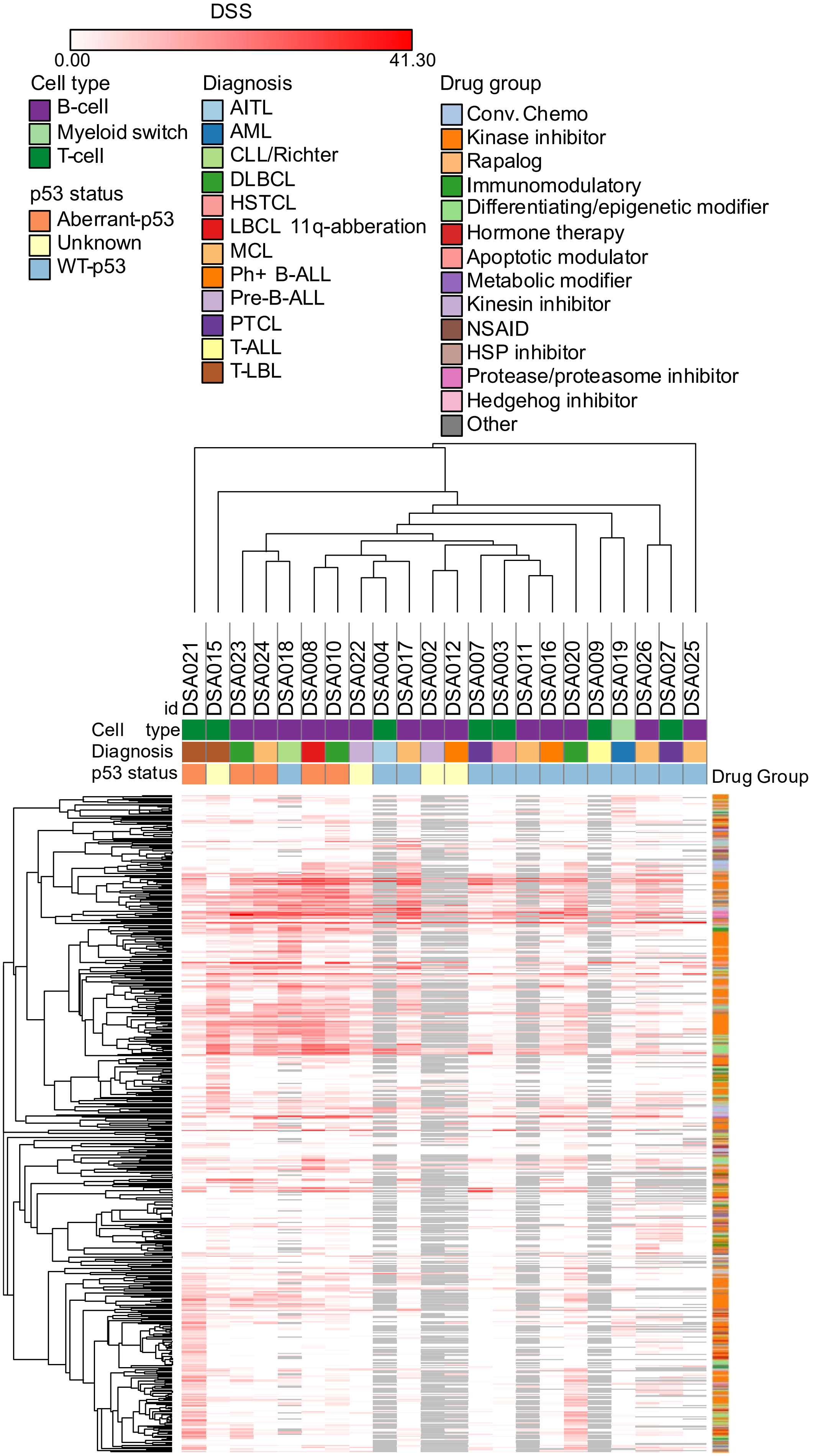

Heatmap of drug sensitivity scores for all drugs in all samples. Gray boxes indicate drugs not tested in the specific patient sample due to 1. Insufficient cell sample for testing or 2. Unavailability of drug at the time of testing. Clustering was done using 1 minus Pearson correlation. AITL = Angioimmunoblastic T-cell Lymphoma, AML = Acute Myeloid Leukemia, CLL = Chronic Lymphocytic Leukemia, DLBCL = Diffuse Large B-cell Lymphoma, HSTCL = Hepatosplenic T-cell Lymphoma, LBCL = Large B-cell Lymphoma, MCL = Mantle Cell Lymphoma, Ph = Philadelphia chromosome, ALL = Acute Lymphoblastic Leukemia, PTCL = Peripheral T-cell Lymphoma, T-LBL = T-Lymphoblastic Lymphoma, sDSS = Selective drug sensitivity score, WT = Wild type.

Supplementary figure 3A-C

A

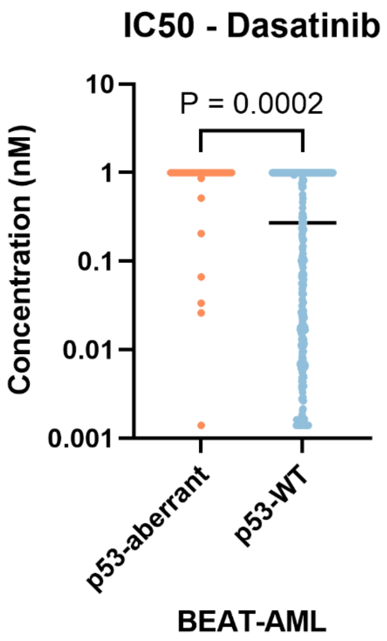

B

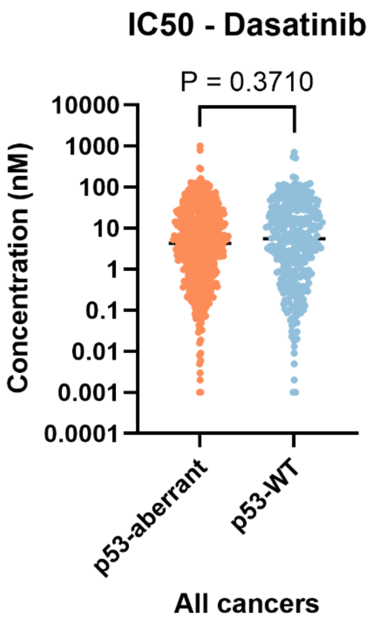

C

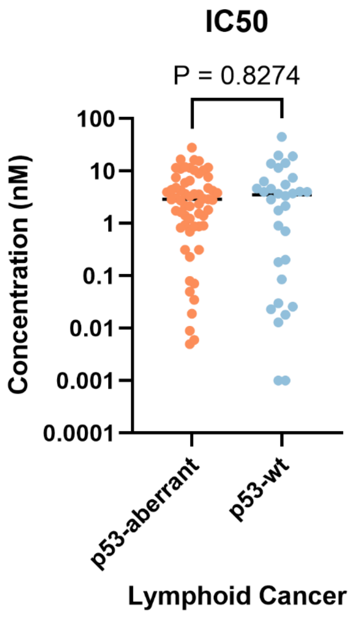

IC50-values for p53-aberrant vs p53-wild type in publicly available material. **A:** IC50 values from AML patients in publicly available data from the BEAT-AML trial. **B:** IC50 values from cell lines across all cancers from DepMap. **C:** IC50 values from lymphoid cancer cell lines from DepMap.

Supplementary data 1 – included as separate excel sheet titled “Supplementary data 1”.

Table showing results from multiple Mann-Whitney U tests for p53-abberant vs p53-wild type sDSS for all tested drugs. FDR (q) correction was set at 1%.

Supplementary data 2 – Included as separate excel sheet titled “Supplementary data 2”

Table of selective drug sensitivity scores of a selection of 50 compounds presented in heatmap 3D. Data is organized with 1 column per sample starting from column D. Missing values are marked with “n/a” and indicate that drugs were not tested in the specific sample due to 1. Insufficient cell sample for testing or 2. Unavailability of drug at the time of testing Abbreviations; AITL = Angioimmunoblastic T-cell Lymphoma, AML = Acute Myeloid Leukemia, CLL = Chronic Lymphocytic Leukemia, DLBCL = Diffuse Large B-cell Lymphoma, HSTCL = Hepatosplenic T-cell Lymphoma, LBCL = Large B-cell Lymphoma, MCL = Mantle Cell Lymphoma, Ph = Philadelphia chromosome, ALL = Acute Lymphoblastic Leukemia, PTCL = Peripheral T-cell Lymphoma, T-LBL = T-Lymphoblastic Lymphoma, WT = Wild type.
